# Is ‘likely pathogenic’ really 90% likely? Reclassification data in ClinVar

**DOI:** 10.1186/s13073-019-0688-9

**Published:** 2019-11-21

**Authors:** Steven M. Harrison, Heidi L. Rehm

**Affiliations:** 1 Medical & Population Genetics Program and Genomics Platform, Broad Institute of MIT and Harvard, Main Street, Cambridge, MA 02142 USA; 2 Department of Pathology, Harvard Medical School, Shattuck Street, Boston, MA 02115 USA; 3 Center for Genomic Medicine, Massachusetts General Hospital, Fruit Street, Boston, MA 02114 USA

## Abstract

In 2015, professional guidelines defined the term ‘likely pathogenic’ to mean with a 90% chance of pathogenicity. To determine whether current practice reflects this definition, ClinVar classifications were tracked from 2016 to 2019. During that period, between 83.8 and 99.1% of likely pathogenic classifications were reclassified as pathogenic, depending on whether LP to VUS reclassifications are included and on how these classifications are categorized.

## Background

In 2015, the American College of Medical Genetics and Genomics (ACMG) and the Association for Molecular Pathology (AMP) published a guideline that provides a framework for sequence variant interpretation [1]. This guideline defined 28 criteria or evidence types, as well as rules for combining these criteria to meet one of the following classification terms for sequence variant interpretation in Mendelian genes: pathogenic (P), likely pathogenic (LP), uncertain significance (VUS), likely benign (LB), or benign (B). Because there is no quantitative definition of the term ‘likely’, the ACMG/AMP committee proposed “that the terms ‘likely pathogenic’ and ‘likely benign’ be used to mean greater than 90% certainty of a variant either being disease causing or benign to provide laboratories with a common, albeit arbitrary, definition” [1]. This committee felt that 90% confidence in pathogenicity was sufficient to warrant physicians taking action, and also high enough that downgrade reclassifications would not be frequent.

A survey of laboratory adoption of the ACMG/AMP guidelines found that 95% of laboratories (62/65 respondents) reported using the ACMG/AMP five tiers to classify variants in Mendelian genes [2]. With regards to the adoption of the evidence criteria provided in the ACMG/AMP guidelines, 97% of laboratories (62/64) reportedly use approaches that are consistent with the guidelines, with 36% using the evidence criteria exactly as described, 44% using an approach that is roughly consistent, and 17% using an approach that they considered a further advancement of the ACMG/AMP approach.

## Variant reclassification

General policies for variant reassessment vary by laboratory. Nevertheless, most laboratories reassess variants when observed in an additional case, at the request of providers, and/or with the release of new interpretation guidelines. Studies focusing on reclassification rates within specific disease areas have shown reclassification rates of between 6.4 and 15%, with this rate being highly dependent on the initial classification type and the date of initial classification. Variants that were initially classified before 2016 show significantly higher reclassification rates than those initially assessed after 2016 [3–5]. Although new evidence, including the emergence of large-scale population databases such as ExAC [6] and gnomAD [7], presumably impacted the relatively high reclassification rate of variants assessed pre-2016, the reduction in reclassification rate also correlates with the release and implementation of the 2015 ACMG/AMP guidelines.

As mentioned above, the 2015 ACMG/AMP guidelines proposed that the term ‘likely pathogenic’ be used to mean greater than 90% certainty of being pathogenic, but no study has analyzed the reclassification of LP variants to determine whether the 90% certainty threshold is being met. Understanding LP classification confidence is necessary because many clinicians treat LP and P classifications equally: their management of care often assumes a causative variant and thus reclassifications to VUS/LB/B are often unanticipated. Given that the majority of laboratories have now adopted the ACMG/AMP guidelines for variant interpretation [2], we sought to determine the true certainty threshold for LP classifications by calculating the reclassification rates of variants submitted to ClinVar [8]. Only variants assessed after 1 January 2016 were included in analyses in the hope of restricting the dataset to those variants classified with the 2015 ACMG/AMP guidelines, but we cannot be certain that all classifications made after this date were based on the 2015 guidelines.

## Analysis of all reclassifications in ClinVar

Between 1 January 2016 and 1 July 2019, 571,850 classifications were submitted to ClinVar using one of the five standard ACMG/AMP classification terms. By 1 July 2019, only 4501 (0.79%) of these classifications had been reclassified by the submitter and updated in ClinVar. Among these reclassifications, 91.9% (4135/4501) moved to a classification category of more certainty (VUS to LP/P, LP to P, VUS to LB/B, LB to B) and only 8.1% (366/4501) moved to either a less certain (7.7%; 347) or an opposing (between P/LP and B/LB; 0.42%; 19) category. Of the five classification terms (Table 1), variants classified as LP had the highest reclassification rate (2.2%; 796).

**Table 1:** Summary of classification and reclassification from ClinVar (Jan 2016–July 2019)

Starting classification (n)	Percentage reclassified (n)	Reclassification type (n)	Percentage of initial classification group	Percentage of all reclassifications
Pathogenic (63,658)	0.17% (110)	P → LP (64)	58.2%	1.4%
		P → VUS (41)	37.3%	0.91%
		P → LB (1)	0.91%	0.02%
		P → B (4)	3.6%	0.09%
Likely pathogenic (36,808)	2.16% (796)	LP → P (625)	78.5%	13.9%
		LP → VUS (165)	20.7%	3.7%
		LP → LB (4)	0.50%	0.09%
		LP → B (2)	0.25%	0.04%
Uncertain significance (272,581)	0.95% (2584)	VUS → P (171)	6.6%	3.8%
		VUS → LP (486)	18.8%	10.8%
		VUS → LB (1586)	61.4%	35.2%
		VUS → B (341)	13.2%	7.6%
Likely benign (140,779)	0.71% (996)	LB → P (2)	0.20%	0.04%
		LB → LP (2)	0.20%	0.04%
		LB → VUS (66)	6.6%	1.5%
		LB → B (926)	93.0%	20.6%
Benign (58,024)	0.03% (15)	B → P (1)	6.7%	0.02%
		B → LP (3)	20.0%	0.07%
		B → VUS (1)	6.7%	0.02%
		B → LB (10)	66.7%	0.22%

*Abbreviations*: *B* Benign, *LB* Likely benign, *LP* Likely pathogenic, *P* Pathogenic, *VUS* Variant of uncertain significance

## Analysis of likely pathogenic reclassifications

Of the 36,808 LP classifications in ClinVar that were annotated as having been assessed after 1 January 2016, 796 were reclassified before 1 July 2019 by the submitting laboratory. Of these 796 LP reclassifications, six were reclassified as LB or B (0.75%), 165 were reclassified as VUS (20.73%), and 625 were reclassified as P (78.52%). Given the absence of a final understanding of the LP to VUS reclassifications (neither pathogenic nor benign/likely benign), we took two approaches to understanding the true LP confidence rate. In the first approach, we only included reclassifications to P or B, that is, we included only those reclassifications that had reached a definitive state. With this conservative approach, LP reclassification rates suggest a 99.7% (625/627) certainty of being pathogenic versus benign. Given that variants that are classified as LB are extremely unlikely to become pathogenic, if LB is included with the B category, this rate is 99.1% (625/631).

A second approach to calculating LP to P reclassification rates incorporated the 20.7% (165/796) of LPs that dropped to VUS, and we used VUS reclassification rates to extrapolate the reclassification rates of ‘LP to VUS’ variants. The current rate of reclassification of VUS variants as LB/B is 74.6% (1927/2584), compared to 25.4% (657/2584) of VUS variants moving to LP/P. If we apply these rates of VUS reclassifications and assume that the same percentage of ‘LP to VUS’ variants will eventually move to LB/B or P/LP, then 25.4% of ‘LP to VUS’ reclassifications would be upgraded to P/LP (42 variants) and 74.6% of ‘LP to VUS’ reclassifications would be downgraded to LB/B (123 variants). Incorporation of these ‘LP to VUS’ extrapolated reclassifications suggests an adjusted LP reclassification rate to P of 83.8% ((625 + 42)/796). However, variants that were initially classified as LP will probably move less frequently to LB/B than variants that started at VUS. Therefore, this extrapolated 83.8% rate is probably an overestimate of the number of ‘LP to VUS’ variants that will be reclassified as LB/B and should be viewed as a ‘worst case scenario’ for the available data.

Variants were further interrogated to determine whether certain variant types were more likely to be upgraded (LP to P) or downgraded (LP to VUS/LB/B). We found that of the predicted loss-of-function (pLoF) LP variants that were reclassified, 88.7% were upgraded to P whereas only 71.1% of missense LP variants that were reclassified were upgraded to P (*p* value < 0.0001; Fisher’s exact test), suggesting that LP confidence is higher for pLOF variants than for missense variants.

To determine trends by disease area, variants in cancer genes and cardiovascular genes from the ACMG secondary findings list [9] were compared. We found that 89.9% (151/168) of LP reclassified cancer variants were upgraded to P, whereas only 75.2% (82/109) of LP reclassified cardiovascular variants were upgraded to P (*p* value = 0.001; Fisher’s exact test). These differences in LP reclassification rates between the two disease groups are probably due to differences in disease mechanism and variant type, because loss of function is the primary mechanism for the majority of cancer conditions on the ACMG secondary finding list, whereas gain-of-function resulting from missense variation is the primary mechanism for many of the cardiovascular conditions [9].

## Conclusions and future directions

In summary, current reclassification data from ClinVar show that 99.7% of LP reclassifications that reached a definitive state moved to P, suggesting that the LP category is being applied consistently with, if not more conservatively than, the 90% definition of pathogenicity. However, the inclusion of reclassifications to LB in the B category suggests a 99.1% rate, and the inclusion of LP reclassifications to VUS (with extrapolation of the final rates of VUS to P and LB/B) suggest an 83.8% rate. A more precise estimate awaits more data on the final classification of the much larger fraction of LPs (97.8%) that currently remain in the LP category. Although the LP category of variants showed the highest rate of reclassification (2.2% of all LP variants), the period of analysis was only a three-and-half year window, and more data and a longer period of analysis will be needed to evaluate the LP reclassifications more robustly. In addition, interrogation of the rationale for LP downgrades could differentiate those resulting from the identification of new evidence from those resulting from reassessment of the original body of evidence. The identification of common issues or scenarios that cause variants to be moved to less certain classifications, as well as further professional and expert guidance on the classification of variants, could increase the confidence and consistency in variant classification. This, in turn, will help to guide physicians in their use of variants classified in the LP category in patient care.

## Data Availability

Variant data are available on ClinVar.

## Abbreviations

ACMG: American College of Medical Genetics and Genomics
AMP: Association for Molecular Pathology
B: Benign
LB: Likely benign
LP: Likely pathogenic
P: Pathogenic
pLoF: Predicted loss of function
VUS: Variant of uncertain significance
